# Busting biofilms: free-living amoebae disrupt preformed methicillin-resistant *Staphylococcus aureus* (MRSA) and *Mycobacterium bovis* biofilms

**DOI:** 10.1099/mic.0.000933

**Published:** 2020-05-27

**Authors:** Kevin H. Martin, Grace I. Borlee, William H. Wheat, Mary Jackson, Bradley R. Borlee

**Affiliations:** ^1^​ Department of Microbiology, Immunology, and Pathology, Colorado State University, Fort Collins, CO, USA; ^2^​ Mycobacteria Research Laboratories, Department of Microbiology, Immunology and Pathology, Colorado State University, Fort Collins, CO, USA

**Keywords:** *Acanthamoeba*, amoebae, biofilm, *Dictyostelium*, *Mycobacterium bovis*, *Staphylococcus aureus*, MRSA, *Vermamoeba*

## Abstract

Biofilm-associated infections are difficult to eradicate because of their ability to tolerate antibiotics and evade host immune responses. Amoebae and/or their secreted products may provide alternative strategies to inhibit and disperse biofilms on biotic and abiotic surfaces. We evaluated the potential of five predatory amoebae – *Acanthamoeba castellanii*, *Acanthamoeba lenticulata*, *Acanthamoeba polyphaga*, *Vermamoeba vermiformis* and *Dictyostelium discoideum* – and their cell-free secretions to disrupt biofilms formed by methicillin-resistant *Staphylococcus aureus* (MRSA) and *Mycobacterium bovis *. The biofilm biomass produced by MRSA and *M. bovis* was significantly reduced when co-incubated with *A. castellanii*, *A. lenticulata* and *A. polyphaga*, and their corresponding cell-free supernatants (CFS). *Acanthamoeba* spp. generally produced CFS that mediated biofilm dispersal rather than directly killing the bacteria; however, *A. polyphaga* CFS demonstrated active killing of MRSA planktonic cells when the bacteria were present at low concentrations. The active component(s) of the *A. polyphaga* CFS is resistant to freezing, but can be inactivated to differing degrees by mechanical disruption and exposure to heat. *D. discoideum* and its CFS also reduced preformed *M. bovis* biofilms, whereas *V. vermiformis* only decreased *M. bovis* biofilm biomass when amoebae were added. These results highlight the potential of using select amoebae species or their CFS to disrupt preformed bacterial biofilms.

## Introduction

A biofilm is a community of bacteria that secrete a matrix consisting of extracellular polymeric substances (EPS), which protects the bacteria adhered to a surface from the environment, host immune responses and other adverse conditions [1, 2]. Bacterial pathogens residing in biofilms exhibit increased tolerance to environmental stressors, such as antibiotics (up to 1000-fold as compared with their planktonic counterparts), nutrient deprivation and temperature flux [3–6]. Biofilms can be especially problematic in hospital settings where patients may be immunocompromised, which increases the risk of infection by commensal microbiota (e.g. *Staphylococcus* spp.) [7, 8]. Bacterial growth in biofilms has contributed to the emergence and spread of antibiotic resistance, which is a public health threat due to the increased prevalence of bacterial pathogens now able to survive current antibiotic treatments, compounded by a decrease in the development of novel antimicrobial drugs [9, 10].

Methicillin-resistant *S. aureus* (MRSA) and *Mycobacterium bovis* are two important public health threats that form robust biofilms, which likely promotes survival in the environment and during infection of a host [11–13]. MRSA has acquired resistance to multiple classes of antibiotics, specifically β-lactams (penicillins and cephalosporins), and is well known for its prevalence in healthcare-acquired infections (HAIs) [14–18]. HAIs that are often associated with biofilm-forming strains of MRSA exhibit a dynamic range of symptoms, which vary from slight skin abrasions to a fatal systemic disease [19, 20]. MRSA residing in biofilms are also more resistant to conventional antimicrobial therapies and host immune responses [16, 21]. *M. bovis* is a slow-growing, Gram-positive aerobe found globally in infected soils and mammalian reservoir species [22–26]. The causative agent of bovine tuberculosis, *M. bovis,* results in tuberculosis-like symptoms in cattle [27, 28] and is the aetiological agent of zoonotic tuberculosis in humans [22, 29, 30]. *M. bovis* biofilm formation on biotic and abiotic surfaces has also been proposed to contribute to drug tolerance, pathogenicity and environmental persistence [31].

There is an urgent need to find alternative strategies to disrupt biofilm-associated infections, which are a contributing factor in increased patient morbidity and mortality [32]. One such strategy is to trigger biofilm dispersal, resulting in the release of planktonic cells that are generally more susceptible to killing by conventional antibacterial treatments [33]. Free-living amoebae (FLA) graze upon attached *Escherichia coli* cells and preformed biofilms produced by a variety of bacteria, including *Klebsiella pneumoniae *, *Pseudomonas* spp. and *Staphylococcus epidermidis* [34–36]. Predatory FLA gain nutrients by engulfing and degrading bacteria and other protists [37] and are also known to secrete molecules into the environment that impact on bacterial survival [38]. For these reasons, amoebal grazing has been hypothesized to be an important factor in controlling biofilms in aquatic environments [39]. Amoebae are single-cell eukaryotes that live as motile trophozoites in nutrient-rich settings [40]. In the presence of stressors or during periods of nutrient starvation, FLA can encyst and produce a double-walled membrane (e.g. *Acanthamoeba* and *Vermamoeba*), thereby becoming extremely resistant to adverse conditions [41, 42].

*Acanthamoeba* are some of the most prevalent amoebae and are widely distributed geographically [43, 44]. Members of the genus *Acanthamoeba* are primarily non-pathogenic but have been reported to opportunistically infect immunocompromised individuals and can also cause amoebic keratitis in contact lens wearers [45, 46]. These amoeba are found in soils and aquatic environments, but also in artificial water systems, including sewers and air-conditioning units [47, 48]. *Vermamoeba vermiformis* is another predatory amoeba found in the environment and has been shown to be a potential reservoir for various pathogens, including *Legionella pneumophila *, *Mycobacterium* spp. and *Stenotrophomonas maltophilia* [49–51]. *Dictyostelium discoideum,* another predatory amoeba [52] found globally in soils, is unique in that it can aggregate into a multicellular slug that can transition into fruiting bodies when challenged with unfavourable conditions [53, 54].

Based on the predatory nature of amoebae and their potential interactions with biofilm-associated bacteria, we hypothesized that MRSA and *M. bovis* biofilms may be altered by amoebae and/or secreted factors produced by amoebae. Three amoebae species – *Acanthamoeba castellanii*, *Acanthamoeba lenticulata* and *Acanthamoeba polyphaga* – and their secretory components prepared from cell-free supernatants (CFS) reduced preformed MRSA biofilms. Furthermore, *A. castellanii*, *A. lenticulata*, *A. polyphaga*, *V. vermiformis* and *D. discoideum,* and their secretory component(s) (excluding *V. vermiformis* CFS) reduced preformed *M. bovis* biofilms. Additional investigation of FLA and their interactions with bacterial biofilms may lead to novel biofilm-disrupting treatments.

## Results

### *Acanthamoeba* spp. and CFS disrupt preformed MRSA biofilms

**Fig. 1.**
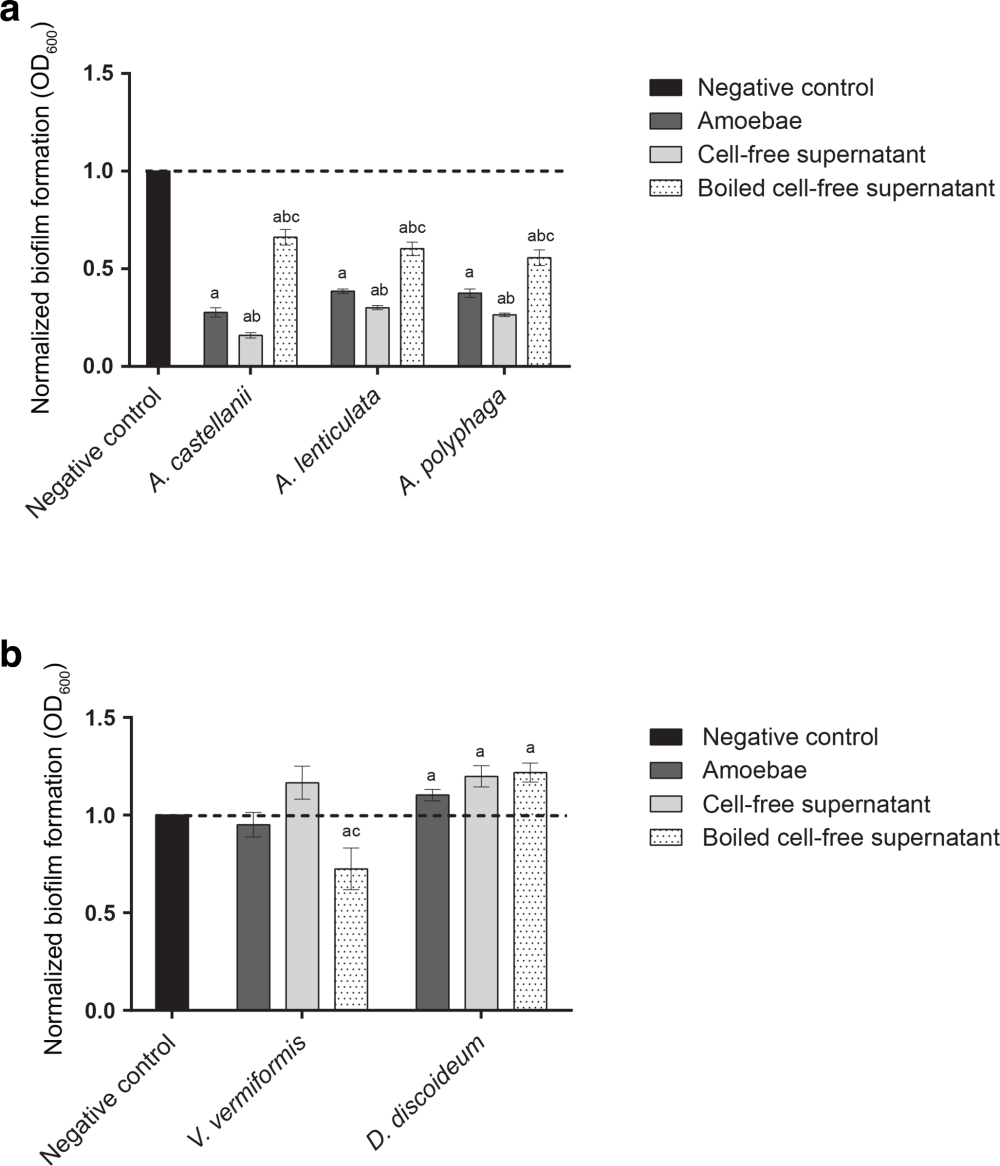
Impact of *Acanthamoeba* spp., *V. vermiformis*, *D. discoideum* and CFS on preformed MRSA biofilms. MRSA biofilm reduction was assessed in the presence of (a) *A. castellanii*, *A. lenticulata* and *A. polyphaga*, and (b) *V. vermiformis* and *D. discoideum* amoebae, CFS and boiled amoebae supernatants after a 24 h incubation period by performing quantitative biofilm biomass assays. These assays consisted of six biological replicates repeated in triplicate. Significance was defined as a calculated *P* value of less than 0.01 by an unpaired Student’s *t*-test (letters indicate significant differences between treatment populations: a, media control; b, to amoebae; c, CFS). Error bars indicate standard error.

Exposure of preformed MRSA biofilms to starved cultures of *A. castellanii*, *A. lenticulata*, or *A. polyphaga* resulted in a significant reduction in biofilm biomass of 72.3, 61.4 and 62.4 %, respectively, as compared to the media control in static biofilm assays (Fig. 1a). Addition of *V. vermiformis* and *D. discoideum* did not decrease MRSA biofilm biomass (Fig. 1b). These results indicate that *Acanthamoeba* spp. differentially reduce MRSA biofilm as compared to the *V. vermiformis* and *D. discoideum* strains tested in this study. MRSA biofilm biomass was further reduced upon co-incubation with CFS obtained from *A. castellanii*, *A. lenticulata* and *A. polyphaga*, which reduced biofilm biomass by 84, 69.9, and 73.6 %, respectively (Fig. 1a). The biofilm-disrupting activity of CFS from *A. castellanii*, *A. lenticulata* and *A. polyphaga* was partially inactivated by boiling. The observed reduction in biofilm biomass from boiled CFS was not as substantial when compared to the reduction observed for co-incubation with amoebae cultures or filtered CFS, suggesting that the biofilm-disrupting activity is only partially heat labile (Fig. 1a). Surprisingly, the addition of *D. discoideum* or its CFS to preformed MRSA biofilms resulted in a significant increase in biofilm biomass of 10.3 and 19.9 %, respectively. The addition of *V. vermiformis* or its CFS to the preformed MRSA biofilms did not alter biofilm biomass, although there was a decrease in biofilm biomass (27.5 %) upon the addition of boiled *V. vermiformis* CFS (Fig. 1b).

**Fig. 2.**
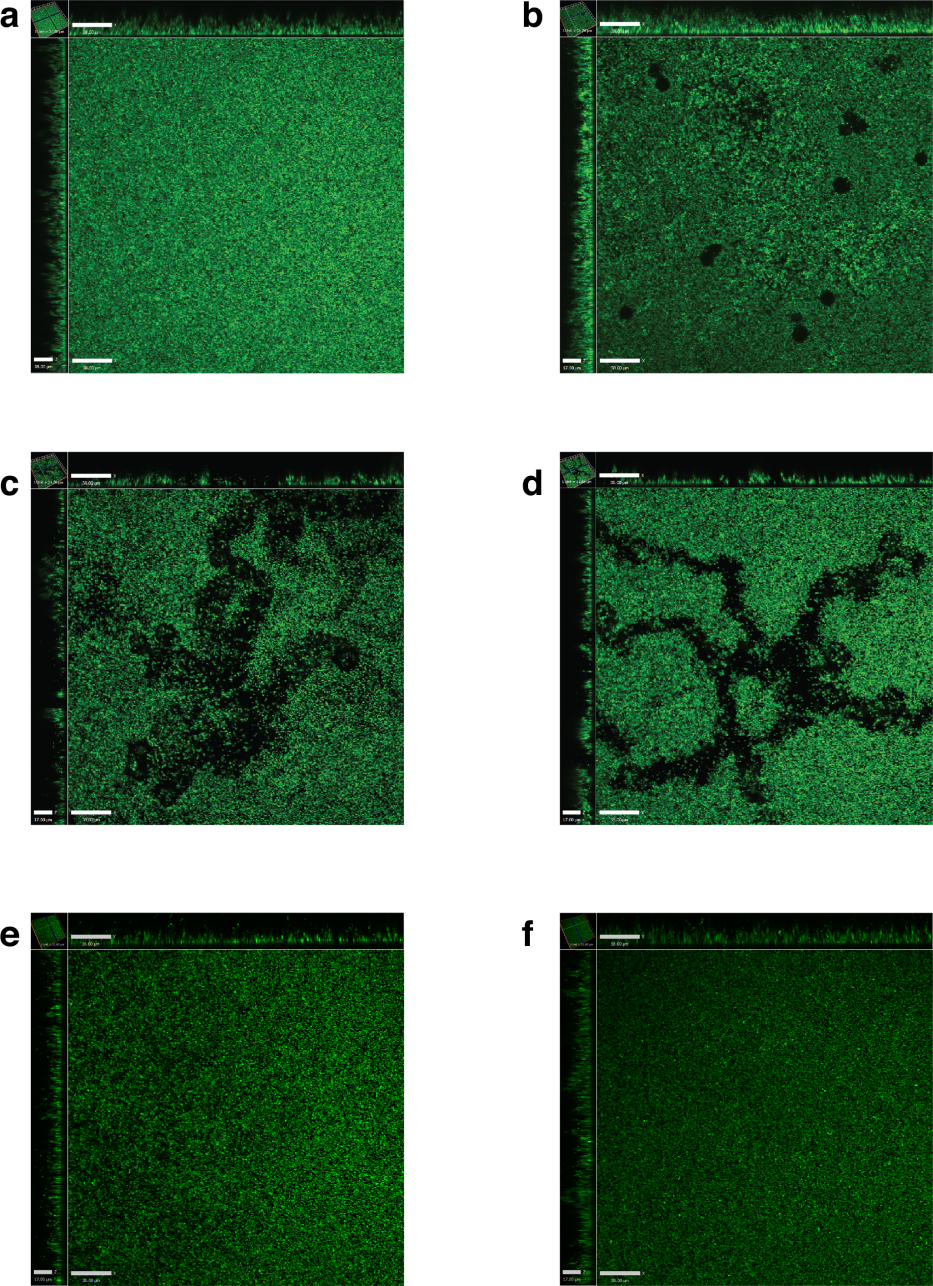
Disruption of preformed MRSA biofilms co-incubated with *Acanthamoeba* spp. Preformed GFP-labelled MRSA biofilms were co-incubated with starved amoebae for 24 h and then subsequently visualized by confocal microscopy. Treatments include a MRSA biofilm (no amoebae control) (a), *A. castellanii* (b), *A. lenticulata* (c), *A. polyphaga* (d), *V. vermiformis* (e) and *D. discoideum* (f). Images shown are representative of results seen across wells. Scale bars: *X*=38.0 µm, *Z*=17.0 µm.

GFP-labelled MRSA cultivated as a biofilm were co-incubated with *A. castellanii*, *A. lenticulata*, *A. polyphaga, V. vermiformis* and *D. discoideum* to visually observe the amoebal interactions with preformed MRSA biofilms. Similar to the biofilm disruption observed in the static biofilm biomass assays, *A. castellanii*, *A. lenticulata* and *A. polyphaga* disrupted the MRSA preformed biofilms. However, the various species of amoebae exhibited different types of behaviours when co-incubated with MRSA biofilms. *A. castellanii* created circular plaques within the MRSA biofilm (Fig. 2b), whereas *A. lenticulata* and *A. polyphaga* bored channels through the biofilm as they actively migrated (Fig. 2c, d; see also Video S1, available in the online version of this article). This observation supports the hypothesis that amoebae may utilize different mechanisms to migrate within and alter bacterial biofilms. The addition of *V. vermiformis* or *D. discoideum* did not produce any observable alterations to the MRSA biofilms. Nearly all the *V. vermiformis* trophozoites encysted upon co-incubation with preformed MRSA biofilms, while *D. discoideum* appeared as non-motile round bodies or floating cells in the presence of the biofilms (Fig. 2e, f).

### *Acanthamoeba* spp. and CFS disperse bacteria from preformed MRSA biofilms

**Fig. 3.**
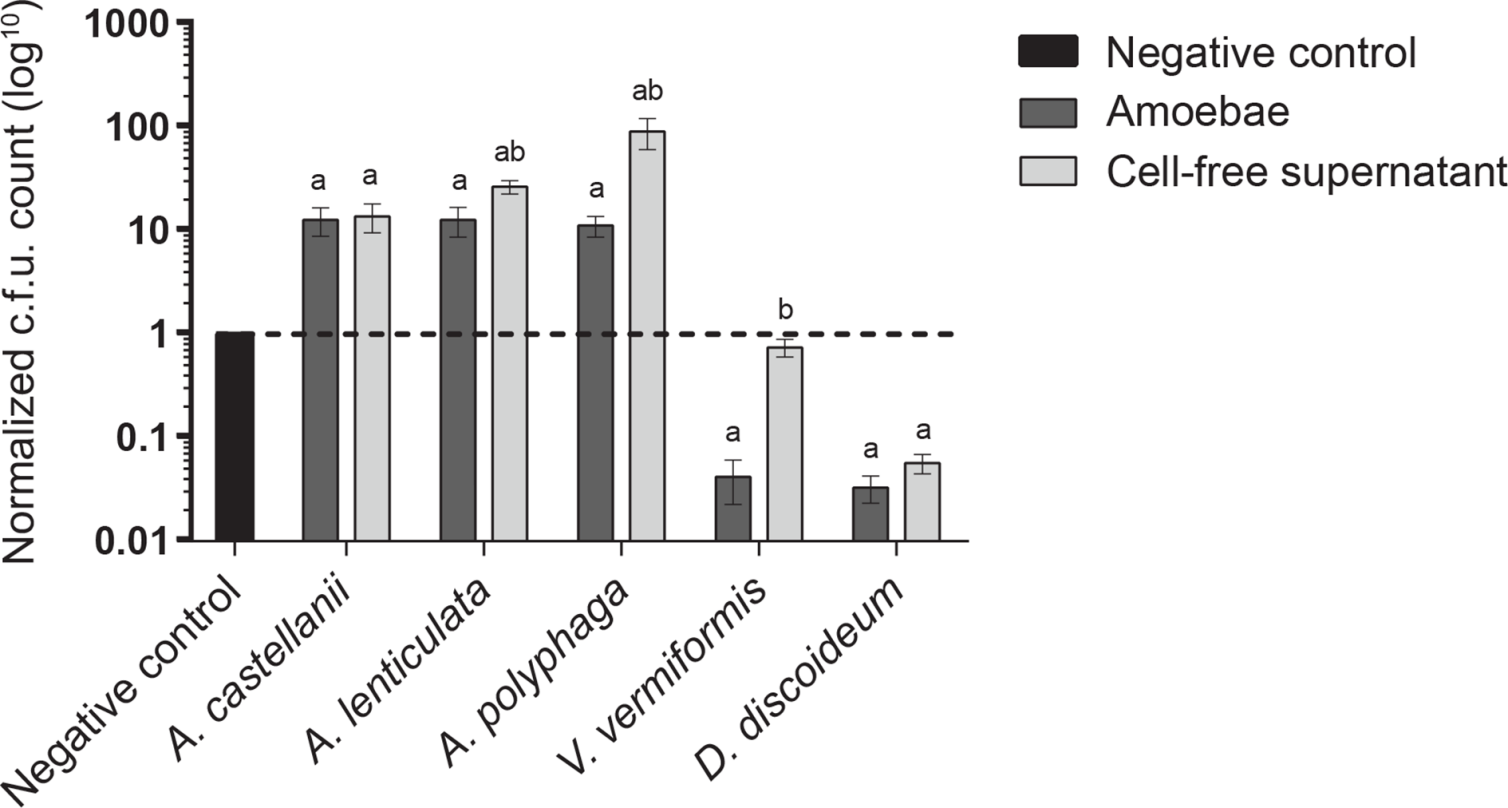
Dispersal of MRSA biofilms co-incubated with *Acanthamoeba* spp., *V. vermiformis* and *D. discoideum* and CFS. Dispersal of MRSA bacteria from preformed biofilms was assessed in the presence of *A. castellanii*, *A. lenticulata*, *A. polyphaga*, *V. vermiformis* and *D. discoideum* and CFS after a 24 h incubation period. Treatments were normalized to a MRSA control biofilm on a log_10_ scale. These assays consisted of six biological replicates repeated in triplicate. Significance was defined as a calculated *P* value of less than 0.01 by an unpaired Student’s *t*-test (letters indicate significant differences between treatment populations: a, media control; b, amoebae). Error bars indicate standard error.

We sought to evaluate whether the primary mode of MRSA biofilm mass reduction upon co-incubation with the amoebae or their CFS resulted from the active killing of the bacteria, dispersal from the biofilm, or a combination of the two. Comparison of colony forming units (c.f.u.) dispersing from preformed MRSA biofilms co-incubated with *A. castellanii*, *A. lenticulata*, or *A. polyphaga* resulted in a significant increase in c.f.u. (127.7, 128 and 112.4%) as compared with the biofilms incubated with the media control (Fig. 3). A significant increase in MRSA c.f.u. was also observed when CFS from *Acanthamoeba* spp. were added to the preformed MRSA biofilms (Fig. 3). The increase in MRSA c.f.u. during co-incubation with the amoebae or their CFS indicates that *Acanthamoeba* spp. evaluated in these studies are actively dispersing the bacteria from the preformed biofilm. Conversely, MRSA was not observed to be dispersing from the biofilm upon the addition of *V. vermiformis, D. discoideum,* or CFS from these amoebae (Fig. 3). The inability of *V. vermiformis* and *D. discoideum* to disperse the MRSA biofilm (Fig. 3) was consistent with data obtained from MRSA biofilm biomass assays and visualization of the structured biofilm communities (Figs 1 and 2). A significant decrease in MRSA dispersal was measured as compared to the media control when MRSA biofilms were co-incubated with *V. vermiformis* and *D. discoideum* (Fig. 3), indicating that these treatments have little effect on disrupting the bacterial biofilm biomass, or may actively reduce planktonic bacterial populations that naturally disperse from the biofilm.

### Activity of *A. polyphaga* CFS on planktonic MRSA

**Fig. 4.**
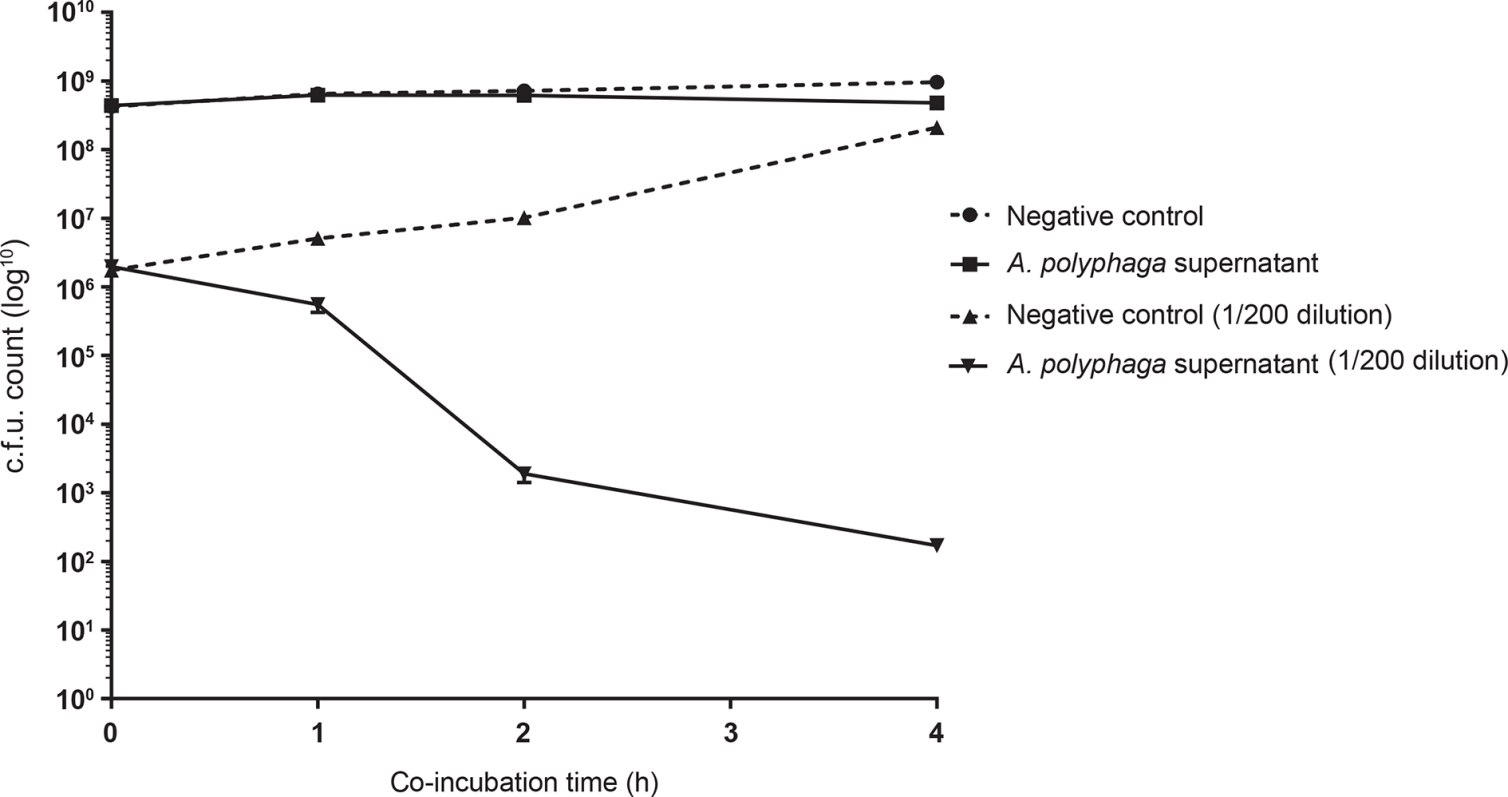
*A. polyphaga* CFS affects planktonic MRSA cells in a concentration-dependent manner. The viability of planktonic MRSA cells was assessed in the presence of *A. polyphaga* CFS at high (OD_600_ 0.6) and 1 : 200 diluted MRSA concentrations on a log_10_ scale. These assays consisted of three biological replicates repeated in triplicate. Significance was defined as a calculated *P* value less than 0.01 by an unpaired Student’s *t*-test. Error bars indicate standard error; some error bars are obscured by their overlapping symbol.

To determine if the *Acanthamoeba* CFS is responsible for killing planktonic MRSA that dispersed from preformed MRSA biofilms, a planktonic MRSA c.f.u. assay was utilized to evaluate whether *A. polyphaga* CFS killed MRSA cells growing as planktonic cells as opposed to within a biofilm. Co-incubation of *A. polyphaga* CFS with MRSA grown to mid-log did not reduce/kill planktonic MRSA as compared with the media controls from 1–4 h post-addition of CFS (Fig. 4). Interestingly, a significant reduction in MRSA was noted when MRSA cells were grown to mid-log, and then diluted 1 : 200 into *A. polyphaga*-conditioned media and co-incubated, as compared with the media controls. Differences in MRSA population density upon co-incubation with *A. polyphaga* CFS may account for whether there is an adverse effect of the active component(s) on planktonic MRSA.

### Initial characterizations of *A. polyphaga* biofilm-dispersing factor(s)

**Fig. 5.**
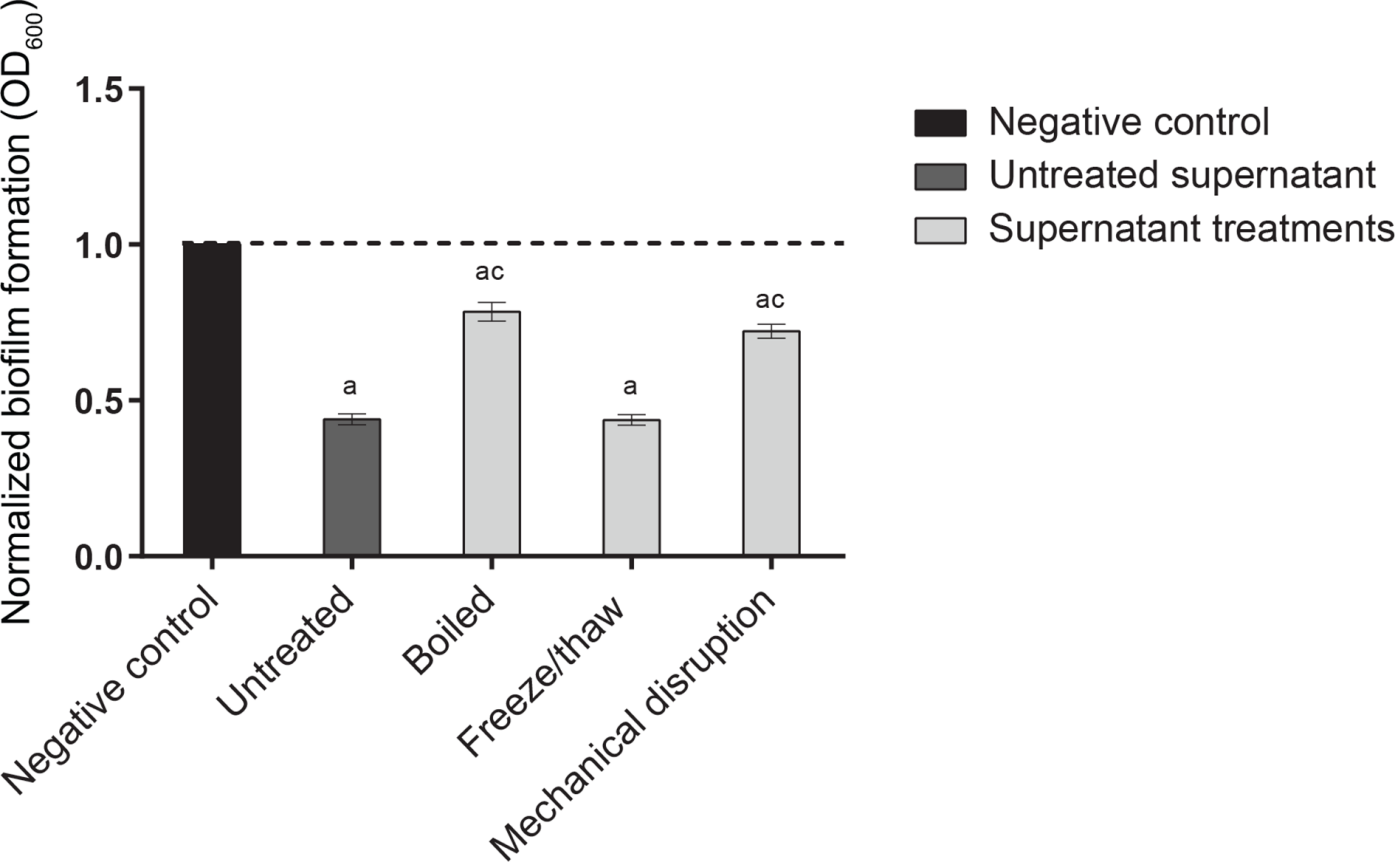
Characterization of the active component(s) in *A. polyphaga* CFS. MRSA biofilm biomass reduction was assessed in the presence of various preparations obtained from *A. polyphaga* CFS after a 24 h incubation period. Treatments include untreated, boiled, freeze/thawed and OmniLyse-treated CFS. These assays consisted of six biological replicates repeated in triplicate. Significance was defined as a calculated *P* value of less than 0.01 by an unpaired Student’s *t*-test (letters indicate significant differences between treatment populations: a, media control; c, cell-free supernatant). Error bars indicate standard error.

*A. polyphaga* CFS was subjected to boiling, mechanical disruption (OmniLyse), or three rounds of freeze/thaw in order to partially characterize the active component(s) responsible for the disruption of preformed biofilms. Inactivation by mechanical disruption may indicate whether extracellular exosomes or vesicles contribute to the active fraction of the supernatant. A significant increase in MRSA biofilm formation was observed when the CFS was either boiled or mechanically disrupted (34.5 and 28.3 %, respectively) as compared with the untreated supernatant (Fig. 5). Boiling and mechanical disruption independently reduced MRSA biofilm biomass (21.6 and 27.8 %, respectively), indicating that the active components of the *A. polyphaga* supernatant are only partially heat-labile and partially sensitive to mechanical disruption. The anti-biofilm component(s) of the *A. polyphaga* CFS was unaffected by three freeze/thaw cycles. (Fig. 5).

### *Acanthamoeba, Vermamoeba* and *Dictyostelium* amoebae and CFS disrupt preformed *M. bovis* biofilms

**Fig. 6.**
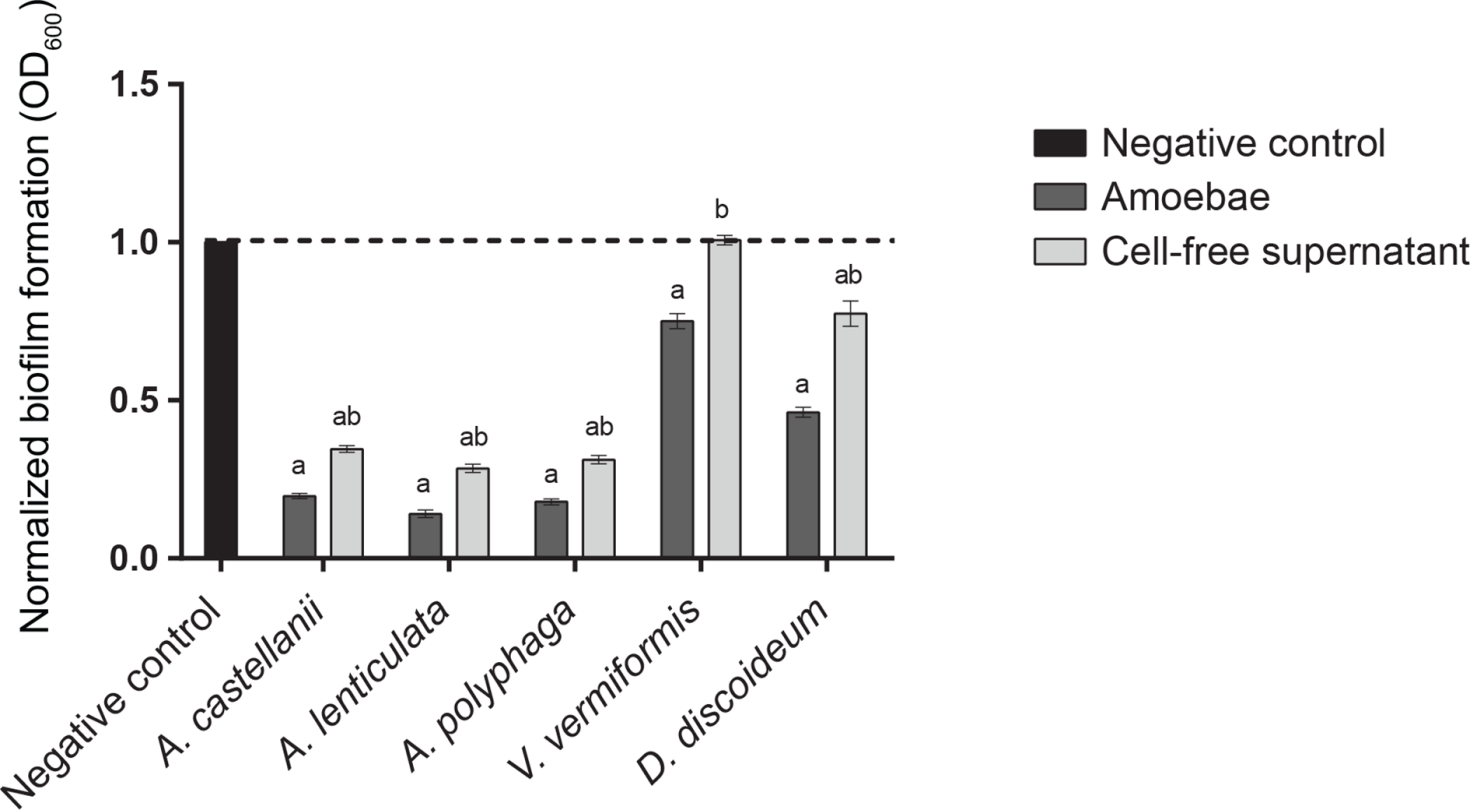
Impact of *Acanthamoeba* spp*., V. vermiformis, D. discoideum* and CFS on preformed *Mycobacterium bovis* biofilms. *M. bovis* biofilm biomass reduction was assessed in the presence of *A. castellanii*, *A. lenticulata*, *A. polyphaga*, *V. vermiformis*, *D. discoideum* and CFS after a 24 h incubation period. These assays consisted of four biological replicates repeated in triplicate. Significance was defined as a calculated *P* value of less than 0.01 by an unpaired Student’s *t*-test (letters indicate significant differences between treatment populations: a, media control; b, amoebae). Error bars indicate standard error.

Co-incubation of preformed *M. bovis* biofilms with *A. castellanii*, *A. lenticulata*, *A. polyphaga*, *V. vermiformis* and *D. discoideum* led to significant decreases in biofilm biomass of 80.3, 85.9, 82.1, 24.9 and 53.8 %, respectively, in the static biofilm assay (Fig. 6). *M. bovis* biofilm biomass was also reduced upon the addition of CFS from *A. castellanii*, *A. lenticulata*, *A. polyphaga* and *D. discoideum* (65.4, 71.5, 68.8 and 22.6 %). No significant change in *M. bovis* biofilm biomass was observed in the presence of *V. vermiformis* CFS.

**Fig. 7.**
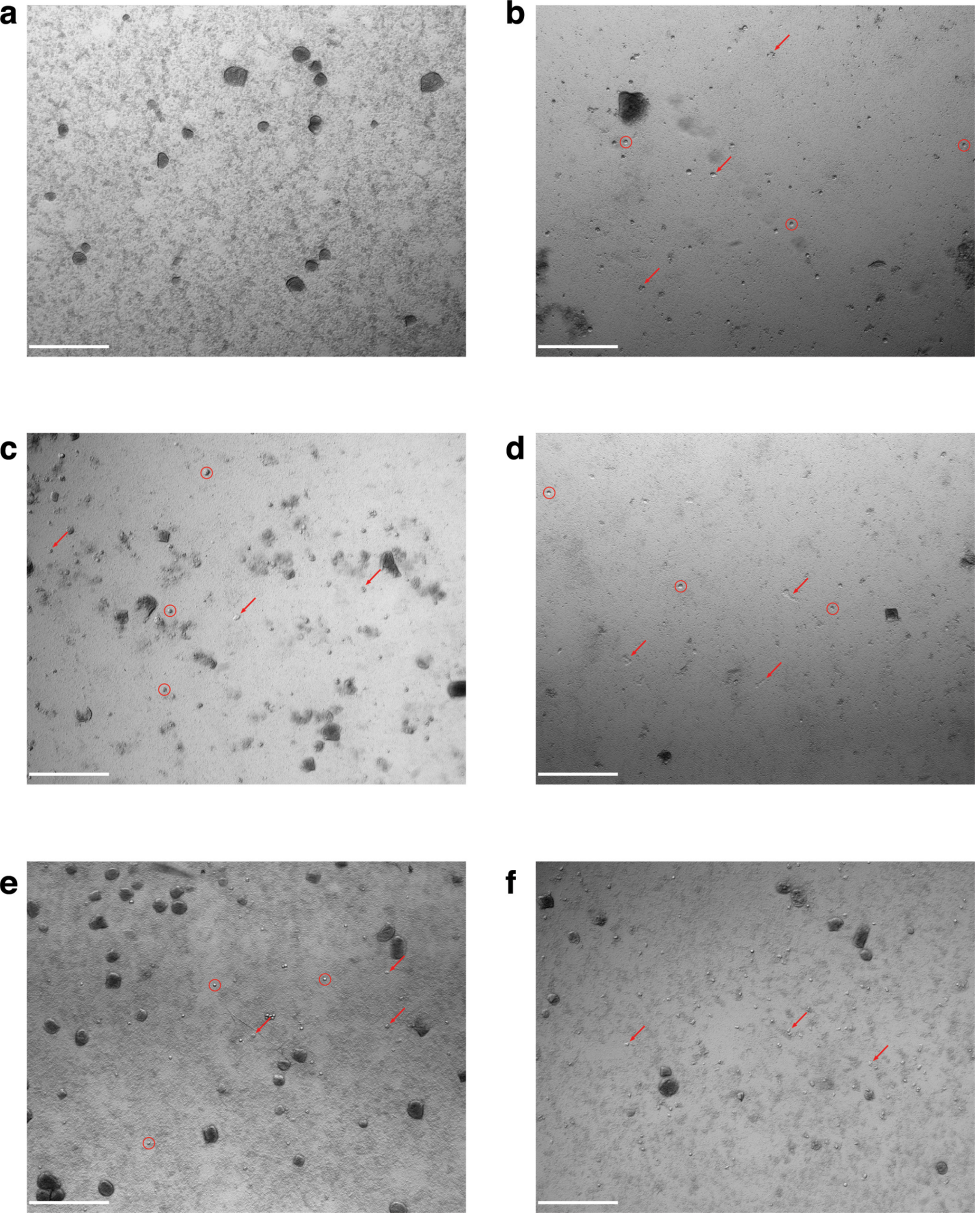
Visualization of *M. bovis* biofilms in the presence of *Acanthamoeba* spp*., V. vermiformis* and *D. discoideum*. *M. bovis* biofilms were visualized for observable alterations in biofilm biomass upon the addition of starved amoebae after a 24 h period of incubation with microscopy. Control *M. bovis* biofilms (a) were performed in parallel with *M. bovis* biofilms exposed to *A. castellanii* (b), *A. lenticulata* (c), *A. polyphaga* (d), *V. vermiformis* (e) and *D. discoideum* (f). Visualizations were completed in duplicate with two biological replicates per assay. Arrows indicate trophozoites whereas circles indicate encysted amoebae. Images shown are representative of results seen across wells. Scale bars indicate 500 µm.

A visible reduction in *M. bovis* biofilm biomass was also observed with microscopy upon co-incubation with *A. castellanii*, *A. lenticulata*, *A. polyphaga*, *V. vermiformis* and *D. discoideum* (Fig. 7a–f). After co-incubation, *A. castellanii*, *A. lenticulata*, *A. polyphaga* and *D. discoideum* had a large proportion of motile trophozoites (red arrows) actively grazing on the preformed *M. bovis* biofilm (Fig. 7b–f), whereas *V. vermiformis* had a large proportion of amoebae encysted (red circles) (Fig. 7b–e). A visible reduction in biofilm biomass by the various amoebae recapitulates the results found in the *M. bovis* biofilm biomass assays, where a significant decrease in *M. bovis* biofilm was observed upon co-incubation with all the amoebae species used in this study (Fig. 6).

## Discussion

In the current study, we sought to characterize the interactions between five different species of amoebae (*A. castellanii*, *A. lenticulata*, *A. polyphaga*, *V. vermiformis* and *D. discoideum*) with preformed biofilms from two pathogenic bacteria, MRSA and *M. bovis *. Amoebae appear to possess mechanism(s) to actively disperse bacteria from a biofilm state, although the details of this mechanism have yet to be determined. Our data demonstrate that *A. castellanii*, *A. lenticulata* and *A. polyphaga* and the CFS that they produce significantly disrupted preformed MRSA and *M. bovis* biofilms. Conversely, *V. vermiformis* and *D. discoideum* and the CFS produced by these amoebae had minimal or no ability to disrupt preformed MRSA biofilms, whereas *V. vermiformis* (amoebae only) and *D. discoideum* amoebae or *D. discoideum* CFS significantly reduced *M. bovis* biofilms. These differential interactions between different species of amoebae and bacteria indicate that there are additional discoveries to be made in this area of research concerning microbe–microbe interactions.

Previous studies have shown that the CFS from *A. polyphaga* inhibits planktonic MRSA growth after 96 h co-incubation [55] and that MRSA can proliferate within *A. polyphaga* [56]. However, untreated and heat-treated *A. castellanii* supernatants killed a significant percentage of planktonic MRSA cells, but did not adversely affect other bacterial species [38]. Bactericidal activity against *Xanthomonas oryzae* pvs. *oryzae* and *oryzicola* by *A. lenticulata*, *A. polyphaga*, *V. vermiformis* and *D. discoideum* has also recently been described [57]. These studies support our observations that *Acanthamoeba* spp. secrete substances into the extracellular environment that can affect specific bacterial species, notably planktonic MRSA cells.

The impact of *A. polyphaga* CFS on planktonic MRSA cells is dependent on the initial concentration and potentially the growth phase of MRSA in the supernatant. When the MRSA population is high (OD_600_ 0.6), the *A. polyphaga* CFS does not significantly reduce the planktonic MRSA population. This finding supports our observation that high populations of bacterial cells from a MRSA biofilm can be dispersed by *Acanthamoeba* spp. without necessarily killing a significant proportion of them in the process. MRSA populations at high cell densities have increased tolerance against antimicrobial factors [21, 58], or may be able to neutralize the active killing components secreted by the amoebae.

Studies addressing amoeba–bacteria relationships can be rather complex. Amoebae have the ability to produce antimicrobial compounds that lyse bacteria [59]; amoebae can also engulf bacteria, resulting in either degradation in lysosomes, or the bacteria may begin intracellular replication. After bacteria are phagocytized, the amoebae may thus serve as a potential reservoir [60, 61]. Alternatively, bacteria have also been shown to produce compounds that induce amoebae encystment or directly kill the amoebae [55, 62, 63]. Amoebae and bacterial interactions are especially complex in the context of bacteria residing in biofilms. In the current study, *Acanthamoeba* spp. actively dispersed cells from both MRSA and *M. bovis* biofilms. Induced dispersal of bacteria by compounds secreted from amoebae presents the amoebae with a plethora of easily accessible planktonic bacteria to graze upon.

Many of the studies involving the identification and characterization of secreted amoebae compounds have focused on the pathogenicity of amoebae to humans and other hosts. The pathogenicity of *Acanthamoeba* spp. can be at least partially attributable to various secreted proteases [64–66], an elastase [67] and a pore-forming toxin [68]. These proteases contribute to amoebae pathogenesis and serve as virulence factors [65, 69], but their role with regards to altering bacterial growth in a biofilm has yet to be determined. Genomic analysis of *A. castellanii* identified an alginate lyase gene that has been predicted to potentially contribute to the degradation of bacterial biofilms composed of the exopolysaccharide alginate produced by *Pseudomonas* spp. [70]. However, the role of this predicted alginate lyase has yet to be characterized. More recently, quantitative proteomic analysis of extracellular vesicles from *A. castellanii* identified hydrolases and oxidoreductases to be the most abundant protein families, comprising roughly 81 % in these vesicles [71].

In the current study, manipulation of *Acanthamoeba* spp. CFS demonstrates that the active component(s) responsible for biofilm disruption are partially heat-labile. Additional manipulation of *A. polyphaga* CFS suggests that the active component(s) are partially inactivated by mechanical disruption (OmniLyse), although the CFS is insensitive to the freeze/thaw process. The observation that activity is lost after mechanical disruption but not after multiple freeze/thaw cycles leads us to hypothesize that the amoebae may be secreting exosomes or extracellular microvesicles (MVs) into the media that can deliver biofilm-dispersing molecules directly to the biofilm [72]. Exosomes and MVs have been described as a means of transferring materials between cells in response to various physiological stimuli [73], but the observation that amoebae may be secreting them for active disruption of bacterial biofilms represents a potentially new mechanism. Further research will focus on understanding and further characterizing the active components of *Acanthamoeba* spp. CFS, which may reveal a novel anti-biofilm compound that can be used in combination with current antimicrobial therapies to treat biofilm-associated infections.

## Methods

### Growth conditions for amoebae

The axenic amoebae used in this study (*A. castellanii* ATCC 30232, *A. lenticulata* ATCC 30841, *A. polyphaga* CCAP 1501/18, *V. vermiformis* ATCC 50237 and *D. discoideum* NC4A1:DBS0236602) were obtained from the American Type Culture Collection and the www.dictybase.org organization. Amoebae were grown as previously described [50], statically in T75 tissue culture flasks in a final volume of 25 ml. *Acanthamoeba* spp. and *V. vermiformis* were maintained at 28 °C, whereas *D. discoideum* was maintained at room temperature (~21 °C). *Acanthamoeba* spp. were grown in 1× PYG45 media by diluting 100 ml of 10× PYG media [200 g l^−1^ proteose peptone (Becton, Dickinson and Company), 20 g l^−1^ yeast extract (Becton, Dickinson and Company), 9.80 g l^−1^ MgSO_4_·7H_2_O (Fisher Scientific), 10 g l^−1^ sodium citrate·2H_2_O, 0.20 g l^−1^ (NH_4_)_2_Fe(SO_4_)_2_·6H_2_O (Sigma-Aldrich), 3.40 g l^−1^ KH_2_PO_4_ (Fisher Scientific), 3.55 g l^−1^ Na_2_HPO_4_·7H_2_O (Fisher Scientific), 90 g l^−1^ α-d-glucose (Fisher Scientific) and 0.59 g/L CaCl_2_ (Sigma-Aldrich)] to 900 ml Page’s amoebae saline (PAS) [60 mg NaCl (Fisher Scientific), 2 mg MgSO4·7H2O (Fisher Scientific), 68 mg KH_2_PO_4_ (Fisher scientific), 71 mg Na_2_HPO_4_ (Sigma-Aldrich) and 2 mg CaCl_2_ (Sigma-Aldrich) (pH = 6.9)]. *V. vermiformis* was grown in modified PYNFH media (ATCC medium 1034) at pH 6.5 [0.059 g ^−1^ peptone (Becton, Dickinson and Company), 10 g l^−1^ yeast extract (Becton, Dickinson and Company), 1 g l^−1^ ribonucleic acid; type VI from torula yeast (Sigma-Aldrich), 15 mg l^−1^ folic acid (Sigma-Aldrich), 1 mg l^−1^ hemin (Sigma-Aldrich), 100 ml^−1^ foetal bovine serum (Peak Serum) and 20 ml ^−1^ buffer solution: 18.1 g l^−1^ KH_2_PO_4_ (Fisher Scientific) and 25 g l^−1^ Na_2_HPO_4_ (Sigma-Aldrich)]. *D. discoideum* was grown in maltose HL5 media (Dictybase.org) at pH 6.65 [14.3 g l^−1^ proteose peptone (Becton, Dickinson and Company), 7.15 g l^−1^ yeast extract (Becton, Dickinson and Company), 18 g l^−1^ maltose monohydrate (Sigma-Aldrich), 0.49 g l^−1^ KH_2_PO_4_ (Fisher Scientific) and 0.641 g l^−1^ Na_2_HPO_4_ (Sigma-Aldrich)]. Cultures were supplemented with 100 U ml^−1^ penicillin/streptomycin (HyClone), passaged every 2–4 days, and discarded after the seventh passage.

*Acanthamoeba* spp. and *V. vermiformis* were starved in 1/5 diluted media before performing assays. Amoebae were first dislodged from the flask using a cell scraper and 5 ml of the culture was centrifuged at 1000 ***g*** for 10 min at room temperature. The supernatant was removed and the pellet was suspended in 10 ml of 1/5 amoebae-specific media, added to a T25 flask and incubated at 28 °C for 24 h. The amoebae were counted using a haemocytometer and trypan blue to assess viability. Cells were diluted to a final concentration of 1×10^5^ amoeba ml^−1^. Amoebae supernatants were passed through a 0.22 µm filter to collect CFS. Supernatants were either untreated, boiled at 110 °C for 30 min, frozen at −80 °C and thawed for three repeats, or treated with an OmniLyse kit (ClaremontBio) for 2 min. Media-only controls were processed in the same manner as the supernatant treatments. Each assay utilizing multiple species of amoebae was taken from the same passage number and the amoebae showed similar levels of growth across the different species.

### Growth conditions for bacteria

MRSA typed as USA300 strains were used for these studies. *S. aureus* strain HFH-29568 [74] was used for all static biofilm assays and obtained from BEI resources (catalogue #NR-10314) through the NIH Biodefense and Emerging Infections Research Resources Repository, National Institute of Allergy and Infectious Diseases (NIAID). A GFP-expressing *S. aureus* strain, AH1726, was used for confocal imaging [75]. MRSA cultures were grown in 22 g l^−1^ Mueller–Hinton II broth (MHB) (Becton, Dickinson and Company) with 10 µg ml^−1^ chloramphenicol (Gold Biotechnology), when necessary, and incubated for 24 h at 37 °C in a shaking incubator. *M. bovis* USDA #95–1315 [76] cultures were grown in 7H9 media supplemented with 0.05 % Tween [4.7 g l^−1^ 7H9 broth base (Becton, Dickinson and Company), 225 µl l^−1^ Tween 80 (Fisher Scientific), 4.1 g l^−1^ sodium pyruvate (Sigma-Aldrich) and 22.5 ml^−1^ Middlebrook OADC Supplement (Fisher Scientific)]. Cultures were started in a 250 ml plastic Erlenmeyer flask and allowed to incubate for 2 weeks in a 37 °C shaking incubator at 150 r.p.m. Cultures were then diluted to a final OD_600_ of 0.1 in modified Sauton’s media at pH 7.0 [0.5 g l^−1^ KH_2_PO_4_ (Fisher Scientific), 0.5 g l^−1^ Mg_2_SO_4_ (Fisher Scientific), 4 g l^−1^ l-asparagine (Sigma-Aldrich), 0.05 g l^−1^ (NH_4_)_5_Fe(C_6_H_4_O_7_)_2_ (Sigma-Aldrich), 2 g l^−1^ citric acid (Fisher Scientific), 4.1 g l^−1^ sodium pyruvate (Sigma-Aldrich) and 100 µl of 1 % ZnSO_4_ solution (Sigma-Aldrich)].

### Static biofilm assay

Bacterial cultures were grown in a 37 °C incubator until turbid (24 h for MRSA and approximately 2 weeks for *M. bovis *) as described above. The cultures were diluted to an OD_600_ of 0.1 and 100 µl was added to the wells of 96-well flat-bottom plates (Nunc Microwell for MRSA and Corning Biocoat poly-d-lysine coated for *M. bovis *). Plates were sealed in a Ziploc bag and incubated at 37 °C (24 h for MRSA and approximately 6 weeks for *M. bovis *). Twenty-four hours before the biofilm was processed, 5 ml of each strain of amoebae was starved as described above. After incubation, the media was removed from the 96-well plate and starved amoebae (or CFS treatments) were added in replicates of 6. Plates were incubated for 24 h at 28 °C for *Acanthamoeba* spp. and *V. vermiformis*, or room temperature (~21 °C) for *D. discoideum*. After incubation, the media were removed from the plate and the plate was rinsed in phosphate-buffered saline (PBS) and then stained with 0.05 % crystal violet for 15 min. The crystal violet was then solubilized in 95 % ethanol for 30 min and absorbance (OD_600_) was measured on either a PerkinElmer EnSpire or a Synergy HT plate reader. These assays were completed in triplicate.

### Quantification of MRSA biofilm dispersal

MRSA cultures were grown for 24 h in a shaking incubator at 37 °C, diluted to a final OD_600_ of 0.1 in MHB, 100 µl of culture was added to Nunc Microwell 96-well microplates, and then the plate was incubated for 24 h at 37 °C in a sealed Ziploc bag. After the 24 h incubation period, MRSA biofilms were exposed to either starved amoebae or amoebae CFS. The supernatant containing cells dispersing from the biofilm was removed from each well and diluted from 10^−1^ to 10^−7^ in PBS and plated onto MHB plates. c.f.u. were counted after 24 h incubation at 37 °C. These assays were completed in triplicate.

### Determination of viability of planktonic MRSA in *A. polyphaga* (CFS)

MRSA cultures were grown for 24 h in a shaking 37 °C incubator, diluted 1 : 50 in 1/5 PYG and placed in a shaking 37 °C incubator until the culture reached an OD_600_ of approximately 0.6 (mid-log). Cultures were added to a 50 ml conical tubes (50 ml total for 1 : 1 dilution and 250 µl total for 1 : 200 dilution) and centrifuged at 4800 ***g*** for 20 min, and then the supernatants were removed. The bacterial pellet was suspended in either 1/5 PYG or *A. polyphaga* CFS, and 3 ml of the culture was added in triplicate to 15 ml plastic tubes. Tubes were placed in a shaking 37 °C incubator and aliquots were plated for c.f.u. in time-increments of 0, 1, 2 and 4 h by dilution from 10^−1^ to 10^−7^ in PBS and plating on MHB plates. c.f.u. were counted after 24 h incubation at 37 °C. These assays were completed in triplicate.

### Confocal imaging

MRSA cultures were grown for 24 h in a shaking 37 °C incubator, the culture was then diluted to an OD_600_ 0.1 in MHB, 300 µl of the culture was added to the wells of a µ-Slide eight-well glass-bottom IBIDI slide (catalogue #80826), and the slides were incubated at 37 °C in a sealed Ziploc bag. After a 24 h incubation period, the media were removed, and the preformed MRSA biofilms were exposed to starved amoebae or control treatments for 24 h. Confocal imaging was performed using an inverted Olympus FV1000-IX81 confocal imaging system. Time-lapse microscopy was obtained with an imaging speed set to 4 µs/pixel, and an imaging interval set to freerun (image acquisitions repeating at approximately 1.6 s/image). The time-lapse video is displayed at 33 frames s^−1^. Images were processed and analysed with Volocity (PerkinElmer). The utilization of IBIDI slides allowed for multiple biological replicates to be visualized simultaneously and the images shown are representative of results seen across multiple treatment wells and control wells. These assays were completed in triplicate.

### Light microscopy

*M. bovis* cultures were grown for 2 weeks in a shaking 37 °C incubator, diluted to a final OD_600_ of 0.1 in modified Sauton’s media, 1500 µl was added to the wells of µ-Slide two-well glass-bottom IBIDI slides (catalogue #80286) coated with poly-d-lysine, and incubated at 37 °C in a sealed Ziploc bag. After an incubation period of approximately 6 weeks, the media were removed and preformed *M. bovis* biofilms were exposed to starved amoebae for 24 h. Imaging was performed using an Olympus IX73 inverted microscope with cellSens software. The images shown are representative of results visualized across treatment and control wells; assays were completed in duplicate.

### Statistical analyses

Statistical analyses were conducted using GraphPad Prism 6. Treatments were normalized to bacterial biofilms grown in media controls; media controls consisted of 1/5 amoebae-specific media subjected to the treatment conditions (i.e. 0.22 µm filtration, boiling, freeze/thaw and mechanical disruption) alongside experimental treatments when necessary. The groups were then analysed using an unpaired Student’s *t*-test. Differences were designated as significant when the calculated *P*-value was less than 0.01. Error bars indicate the standard error.

## Supplementary Data

Supplementary material 1
